# Relationship of Inter-Arm Systolic Blood Pressure Difference with Subclavian Artery Stenosis and Vertebral Artery Stenosis in Patients Undergoing Carotid Endarterectomy

**DOI:** 10.21470/1678-9741-2018-0257

**Published:** 2019

**Authors:** Serkan Burç Deşer, Semih Murat Yucel, Mustafa Kemal Demirag, Fersat Kolbakir, Hasan Tahsin Keceligil

**Affiliations:** 1 Department of Cardiovascular Surgery, School of Medicine, Ondokuz Mayis University, Samsun, Turkey.

**Keywords:** Subclavian Steal Syndrome, Carotid Stenosis, Blood Pressure Determination/Methods, Carotid Endarterectomy, Systole

## Abstract

**Introduction:**

The aim of this study was to examine the association of inter-arm systolic blood pressure difference (IASBPD) with carotid artery stenosis, subclavian artery stenosis and vertebral artery stenosis in patients who underwent carotid endarterectomy.

**Methods:**

A total of 141 patients (29 females, 112 males; mean age 71.2±10.4 years; range 47 to 92 years) who underwent carotid endarterectomy between September 2010 and December 2017 were retrospectively evaluated. We classified patients into four groups according to the IASBPD ˂ 10 mmHg, ≥ 10 mm Hg, ≥ 20 mmHg and ≥ 30 mmHg. The stenosis of both subclavian and vertebral arteries was considered as ≥ 50%.

**Results:**

Of the 141 patients, 44 (31.2%) had ≥ 10 mmHg, 29 (20.5%) had ≥ 20 mmHg and 4 (2.8%) had ≥ 30 mmHg of IASBPD. 26 patients (18.4%) were diagnosed with significant subclavian artery stenosis and 18 (69.2%) of them had more than 20 mmHg of IASBPD. Of the 29 patients with IASBPD ≥ 20 mmHg, 19 patients (65.5%) had a significant subclavian artery stenosis. We found a significant correlation between preoperative symptoms and subclavian artery stenosis (*P*=0.018) and overall perioperative stroke was seen more frequently in patients with subclavian artery stenosis (*P*=0.041). A significant positive correlation was observed between vertebral artery stenosis and subclavian artery stenosis (*P*=0.01).

**Conclusion:**

Patients who were diagnosed with both subclavian artery stenosis and IASBPD (≥ 20 mmHg) had a higher risk of postoperative stroke and death, had higher total cholesterol, LDL-C, blood creatinine level, and were more symptomatic.

**Table:** 

Abbreviations, acronyms & symbols				
CEA	= carotid endarterectomy		ICU	= Intensive care unit
ESC	= European Society of Cardiology		IQR	= Interquartile range
ESH	= European Society of Hypertension		LDL-C	= Low density lipoprotein cholesterol
FT3	= Free T3		NASCET	= North American Symptomatic Carotid Surgery Trial
FT4	= Free T4		RR	= Relative risk
HB	= Hemoglobin		SAS	= Subclavian artery stenosis
HDL-C	= High density lipoprotein cholesterol		SD	= Standard deviation
IASBPD	= Inter-arm systolic blood pressure difference		SPSS	= Statistical Package for the Social Sciences
ICA	= Internal carotid artery		TSH	= Thyroid-stimulating hormone

## INTRODUCTION

An increased inter-arm systolic blood pressure difference (IASBPD) is usually considered as ≥ 10 mmHg, and the reported prevalence of the population ranges between 3,6% and 9.4%^[1-3]^. IASBPD over 10 mmHg of is usually associated with subclavian artery stenosis, cerebrovascular disease, stroke, cardiovascular morbidity and mortality, atherosclerosis and left ventricular hypertrophy^[1-3]^. Additionally, IASBPD over 15 mmHg constitutes a 1,6 fold risk of cardiovascular morbidity and mortality^[4-11]^. Perioperative uncontrolled hypertension is observed in 13-23% of patients undergoing carotid endarterectomy (CEA) and associated with perioperative stroke and death^[12,13]^. For this reason, being aware of IASBPD is essential for the appropriate treatment of perioperative hypertension, especially in patients who underwent carotid endarterectomy^[14]^. The aim of this study was to examine the prevalence, association and outcomes of IASBPD, subclavian artery stenosis and vertebral artery stenosis in patients undergoing carotid endarterectomy.

## METHODS

One hundred and forty-one patients (29 females, 112 males; mean age 71.2±10.4 years; range 47 to 92 years) who underwent carotid endarterectomy between September 2010 and December 2017 were retrospectively included in this historic prospective study. Treatment indication for symptomatic patients was ≥ 50% stenosis, and for asymptomatic patients was ≥ 70% stenosis of the internal carotid artery, according to the North American Symptomatic Carotid Surgery Trial (NASCET) criteria^[15]^. All demographic and clinical datas of the patients were retrieved from the hospital database. Six patients with combined CEA with coronary artery bypass grafting surgery and five patients with incomplete data were excluded from this study. Systolic and diastolic blood pressures were measured non-invasively at the time of admission and invasively monitored during the surgery and after the surgery in the intensive care unit. We divided patients into four groups according to the IASBPD as; <10 mmHg, ≥ 10 mm Hg, ≥ 20 mmHg and ≥ 30 mmHg^[16]^. Furthermore, patients were divided into another two groups with respect to significant stenosis of the subclavian artery (≥ 50%) and the vertebral artery (≥ 50%)^[17]^.

The internal carotid artery stenosis was initially diagnosed with color doppler ultrasonography and then the stenosis of carotid, subclavian and vertebral arteries were proven by computed tomography angiography, according to the NASCET criteria^[15]^. Additionally, routine transthoracic echocardiographic was performed on all patients in order to decide preoperative coronary angiography according to left ventricle ejection fraction and motion disorder of left ventricle wall. Patients' initial symptoms and physical examination, risk factors, laboratory analysis, perioperative complications, length of intensive care unit/in-hospital stay were analyzed. Conventional longitudinal endarterectomy with dacron or saphenous patch closure or modified eversion endarterectomy techniques were performed to all patients. Perioperative major neurological complications were considered as hemiplegia, hemiparesis, transient ischemic attack, and cerebral hyperperfusion syndrome. All patients undergoing surgery were under single antiplatelet therapy (acetic salicylic acid 100 mg/day or clopidogrel 75 mg/day) and continued with a single or dual antiplatelet therapy throughout their life, and low molecular weight heparin was administered for 3 days after the surgery. Cranial computed tomography scan, diffusion-weighted magnetic resonance imaging and carotid color doppler ultrasonography were performed in case of postoperative stroke.

The regional ethical committee approved the study (OMU KAEK 2017/353). The study was carried out in accordance with the Helsinki Declaration principles.

### Statistical Analysis

The Statistical Package for the Social Sciences Windows Version 21 (SPSS Inc, Chicago, IL, USA) was used to compare the datas. The Kolmogorov-Smirnov test was used to analyze normally distributed continuous variables. Categorical variables were presented in percentages and frequencies. Continuous variables were presented as the mean ± standard deviation (SD). Independent sample t-tests were used to compare the means of dependent groups. The continuous variables were compared using the t-test and the Mann-Whitney U test. The categorical datas were tested with the chi-square test or Fisher's exact test. A *P*-value of <0.05 was considered statistically significant.

## RESULTS

Of the 163 patients, perioperative blood pressure measurement data were available in 141 patients. The mean inter-arm systolic blood pressure difference was 11,23 mm Hg (range 0-40 mm Hg). 44 patients (31.2%) had an IASBPD ≥ 10 mmHg, 29 patients (20.5%) had an IASBPD ≥ 20 mmHg, and 4 (2.8%) patients had an IASBPD of ≥ 30 mmHg, while IASBPD was detected lower than 10 mmHg in 97 patients (68.7%). Table 1 summarizes the baseline characteristics of patients with IASBPD over 20 mmHg and under 20 mmHg. Male gender was not associated with over 20 mm Hg of IASBPD (*P*=0.89) and no correlation was detected between IASBPD and left or right internal carotid artery stenosis (*P*=0.63, *P*=0.78, respectively) and treated internal carotid artery site (*P*=0.24). However, we found a significant correlation between patients with IASBPD over 20 mmHg and ipsilateral subclavian artery stenosis (*P*=0.009).

**Table 1:** Preoperative comparison of patients with Inter-arm systolic blood pressure difference (IASBPD) more than 20 mmHg and patients with IASBPD less than 20 mmHg who underwent carotid endarterectomy.

	Patients with IASBPD≥20 mmHg (n=29)			Patients with IASBPD < 20 mmHg (n=112)			*P*
	n	%	Mean±SD	n	%	Mean±SD	
Gender							
Male	23	79.3		90	80.3		0.89
Surgical Side							
Right	17	58.6		67	59.8		0.90
Higher systolic blood pressure side							
Right arm	15	51.7		63	56.2		0.66
Left arm	4	13.7		23	20.5		0.41
Symptomatic	5	17.2		23	20.5		0.69
Asymptomatic	24	82.8		89	79.5		0.69
Body mass index			26.7±3.7			26.9±4.3	0.81
Current smoking	5	17.2		24	21.4		0.61
Diabetes mellitus	19	65.5		69	61.6		0.69
Hypertension	17	58.6		60	53.5		0.62
Coronary artery disease	8	27.5		34	30.3		0.77
Peripheral artery disease	4	13.7		21	18.7		0.78
Chronic obstructive pulmonary disease	3	10.3		20	17.8		0.40
Lipid lowering therapy	9	31		30	26.7		0.64
Antithrombotic therapy	12	41.3		45	40.1		0.90

IASBPD=inter-arm systolic blood pressure difference; SD=standard deviation

Of the 141 patients, 26 patients (18.4%) were diagnosed with significant subclavian artery stenosis, while no significant stenosis was detected in 115 patients (81.6%). 5 patients (3.5%) had bilateral subclavian artery stenosis. Of the 29 patients with IASBPD ≥ 20 mmHg, 19 patients (65.5%) had a significant subclavian artery stenosis (*P*=0.001). Of the 26 patients with significant subclavian artery stenosis, 18 patients (69.2%) had more than 20 mmHg of IASBPD (*P*=0.001) and no IASBPD was detected in 5 patients (19.2%). 47 patients (29%) (15 patients with subclavian artery stenosis, 32 patients without subclavian artery stenosis) were symptomatic. A significant correlation was found between preoperative symptoms and subclavian artery stenosis (*P*=0.018), in other words, patients with subclavian artery stenosis were more symptomatic. The mean IASBPD in patients with subclavian artery stenosis was 20.5±5.3 mm Hg and the mean IASBPD in patients without subclavian artery stenosis was 8.5±3.1 mm Hg (*P*=0.01). Patients with subclavian artery stenosis had higher total cholesterol level (*P*=0.01), higher LDL-C level (*P*=0.015), higher blood creatinine level (*P*=0.017), higher free T3 level (*P*=0.022), higher postoperative-day 1 systolic blood pressure (*P*=0.023), were older (*P*=0.045), and ICU/overall in-hospital stay differences were longer (*P*=0.01 and *P*=0.01, respectively). Additionally, no systolic blood pressure difference was noted in terms of subclavian artery stenosis (149,8 mmHg *vs*. 146,4 mmHg, *P*=0.78). Table 2 compares patients with subclavian artery stenosis and without who undergoing carotid endarterectomy.

**Table 2:** Comparison of patients with subclavian artery stenosis (SAS) and without SAS who underwent carotid endarterectomy.

	Patients with SAS (n=26)			Patients without SAS (n=115)			*P*
	n	%	Mean±SD	n	%	Mean±SD	
Age (year)			73.6±4.6			69.5±10.1	0.045*
Symptomatic	15	57.6		32	27.8		0.018*
HbA1c (NGSP)			6.5±1.7			6.4±1.4	0.75
Hb (g/dL)			14.7±1.7			14.9±1.5	0.55
Htc (%)			43.8±4.6			44.6±4.1	0.38
Total cholesterol (mg/dL)			226.4±16.94			205.3±20.37	0.01*
Triglycerides (mg/dL)			152.2±75.2			160.1±86.3	0.66
HDL-C (mg/dL)			41.6±11.9			40.3±11.2	0.59
LDL-C (mg/dL)			164.56±32.4			153.4±17.54	0.015*
Blood creatinine (mg/dL)			1.68±1.34			1.23±0.72	0.017*
fT3 (pg/mL)			3.13±1.11			2.7±0.79	0.022*
fT4 (ng/dL)			1.33±0.26			1.4±0.45	0.44
TSH (mIU/mL)			1.7±1.4			1.84±3.2	0.82
ICU stay (days)			11.2±7.5			1.2±0.45	0.01*
Overall in-hospital stay (days)			28.2±18.3			3.1±0.71	0.01*
Perioperative stroke							
Thrombo-embolic	2			-			0.032*
Cerebral hyperperfusion syndrome	2			-			0.032*
ICA occlusion	-			1			1
Overall	4			1			0.041*

* Significant

fT3=free T3; fT4=free T4; Hb=hemoglobin; HDL-C=high density lipoprotein cholesterol; Htc=hematocrit; ICA=internal carotid artery; ICU=intensive care unit; LDL-C=low density lipoprotein cholesterol; SAS=Subclavian artery stenosis; SD=standard deviation; TSH=thyroid-stimulating hormone

The median follow-up time was 4,6 years (IQR, 2.0-5.0, maximum 8 years). In 80 patients, (56.7%) right arm systolic blood pressure was higher than the left arm while, in 29 patients (20.6%) left arm systolic blood pressure was higher, and 32 patients (22.7%) had no systolic blood pressure difference between arms. 31 patients (21.9%) had vertebral artery stenosis (15 right, 10 left and 6 bilateral). Of the 26 patients with subclavian artery stenosis, 16 patients (61.5%) were accompanied with vertebral artery stenosis (*P*=0.01). However, no significant correlation was found between vertebral artery stenosis and, stroke and carotid artery stenosis (*P*=0.12 and *P*=0.23, respectively).

A perioperative stroke had occurred in 5 patients (3.5%). The most common reason of perioperative stroke was comprising thrombo-embolic event in 2 patients (1.4%), cerebral hyperperfusion syndrome in 2 patients (1.4%) and internal carotid artery occlusion in 1 patient (0.7%). 4 patients (2.8%) were diagnosed with both subclavian artery stenosis and IASBPD (≥ 20 mmHg) (*P*=0.04) and 1 patient (1.4%) neither had subclavian artery stenosis nor IASBPD. Post operative death occurred in 3 patients (2.1%) with both subclavian artery stenosis and IASBPD (*P*=0.0057, *P*=0.008, respectively), while no perioperative myocardial infarction occurred due to our strict cardiac examination.

## DİSCUSSİON

This historic prospective study demonstrates that patients who were diagnosed with both subclavian artery stenosis and IASBPD (≥ 20 mmHg) had a higher risk of perioperative stroke and death, higher total cholesterol, LDL-C and blood creatinine level, and were more symptomatic. In addition, subclavian artery stenosis was associated with vertebral artery stenosis. To the best of our knowledge, this is the first study to evaluate the relationship between IASBPD and carotid artery stenosis, subclavian artery stenosis and vertebral artery stenosis in terms of perioperative outcomes in patients undergoing carotid endarterectomy.

Perioperative blood pressure disturbances are seen in 13-23% of patients undergoing carotid endarterectomy^[13,18]^. Thus, perioperative blood pressure measurement of both arms is essential to avoid mistaken blood pressure measurement, which may lead to perioperative stroke due to cerebral hyperperfusion syndrome in patients undergoing carotid endarterectomy^[12,13,17,19,20]^. The relation of subclavian artery stenosis and more than 20 mmHg of IASBPD is already well established with a recent meta-analysis conducted by Cao et al.^[2]^ , which supported this strong association (RR 8.8, 95% CI 3.6-21.2, *P*<0.01) and our findings were consistent with this previous study (*P*=0.009). English et al.^[20]^reported that the sensitivity and the specificity to detect the subclavian artery stenosis for more than 10 mmHg of IASBPD was 65% and 85% while, 20 mmHg of difference was 35% and 94%, respectively. Worthy of note, most of the previous studies considered a threshold value of 20 mmHg^[17,19]^ thus we considered 20 mmHg of IASBPD as a threshhold value.

Huibers et al.^[17]^published a 182-patient, multicenter, randomized control trial where 20% of the patients had more than 15 mmHg of IASBPD. Of those patients, 20% of them were accompanying with subclavian or innominate artery stenosis, in patients undergoing carotid endarterectomy. In addition, Huibers et al.^[17]^stated that 47.6% of patients with subclavian artery stenosis had more than 10 mmHg of IASBPD while, 23.3% of patients with more than 20 mmHg of IASBPD had a significant subclavian artery stenosis. Our findings were higher than the previous study, where 69.2% of patients with subclavian artery stenosis had more than 20 mmHg of IASBPD, while 65.5% of patients with more than 20 mmHg of IASBPD had a significant subclavian artery stenosis. The incidence of both subclavian artery stenosis and more than 20 mmHg of IASBPD was 94.7%^[18,19]^. We concluded that these relatively high ratios depend on the routine measurement of both arm systolic blood pressure in our cardiovascular surgery clinic. In addition, computed tomography angiography was routinely performed to all patients diagnosed with severe stenosis by color doppler ultrasonography, and both subclavian and vertebral arteries were examined simultaneously.

Kranenburg et al.^[19]^reported a strong relation with IASBPD and the carotid artery stenosis in patients with peripheral vascular diseases, whereas we did not note any correlation (*P*=0.78). Besides that, no superiority was found for each arm systolic blood pressure in regard to outcomes^[19]^. In this context, our study demonstrated a similar manner with previous studies ^[17,19]^, that no superiority of left and right arm systolic blood pressure was detected (*P*=0.672).

Huibers et al.^[17]^reported that the prevalence of patients with bilateral subclavian artery stenosis was 1%. However, 3.5% of patients (5 patients) had bilateral subclavian artery stenosis in our study. Of note, systolic blood pressure difference can not be detected between both arms in case of bilateral subclavian artery stenosis. Although, we did not find any relations between subclavian artery stenosis and carotid artery stenosis, it is interesting to note that, in the present study, a significant correlation was found between preoperative symptoms and subclavian artery stenosis (*P*=0.018). In other words, the majority of symptomatic patients were diagnosed with subclavian artery stenosis at the same time. In addition, total cholesterol, LDL, blood creatinine level, fT3 level, postoperative day 1. systolic blood pressure was higher and ICU/overall in-hospital stay was longer in patients with subclavian artery stenosis, and patients were older. We thought that atherosclerosis was more advanced in those patients and significant disease was also present in all systemic arteries except carotid arteries. This mechanism might also explain why we found a higher ratio of subclavian artery stenosis in our study, contrary to Huibers et al.^[17]^. Therefore, in those patients, blood pressure should be measured in both arms and further examination should be performed in terms of subclavian artery stenosis.

In patients with carotid artery stenosis, aortic arches and branches are routinely examined, so an additional imaging modality is not required in the diagnosis of subclavian artery stenosis and vertebral artery stenosis without excessive radiation exposure to patients. In this study, the proportion of patients with IASBPD greater than 10 mmHg was found to be relatively low (31.2%) among all patients. When we increased the threshold level from 10 mm Hg to 20 mm Hg, this rate decreases to 20.5% of patients.

We found a significant correlation between subclavian artery stenosis and vertebral artery stenosis, thus we re-evaluated patients in terms of preoperative vertebra-basilary system symptoms such as dizziness. However, no difference was found as expected in symptomatic patients. In addition, no subclavian artery occlusion, nor subclavian steal phenomenon were noted in any patient, although the rate of significant subclavian artery stenosis was found higher rather than previous studies^[21]^.

The relation between subclavian artery stenosis and atherosclerosis is obvious. In this current study, the perioperative stroke was seen more frequently in patients with subclavian artery stenosis (*P*=0.041). This mechanism might also explain by cerebral hyperperfusion syndrome due to mistaken systolic blood pressure measurement or atherosclerosis level may be more advanced in those group of patients with a worse vascular structure (*P*=0.032). In addition, Huibers et al.^[21]^reported that IASBPD was existing in all patients who had a perioperative stroke due to cerebral hyperperfusion syndrome.

There are some limitations of our study. First, this study had a retrospective design. Second, the number of patients in our study may seem limited compared with other studies. Third, larger studies would be required to replicate our findings. Fourth, the power of some outcomes may have been reduced due to this single-center study. Fifth, IASBPD may change in subsequent years due to a relatively short follow-up period of 4.6 years. Sixth, as mentioned above, we thought that patients with subclavian artery stenosis had more severe atherosclerosis level; however, we did note any correlation with peripheral arterial disease, this issue should be focused on further prospective studies. Seventh, we did not compare patients with 20-30 mmHg of IASBPD and patients with more than 30 mmHg of IASBPD, owing to a low volume of patients who had more than 30 mmHg of IASBPD. Strengths of this study include the measuring both arm blood pressures in all patients during admission and computed tomography angiography was performed to all patients. However, our results might need further validation in community-based cohorts.

## CONCLUSİON

Our findings in this single-center study suggest that measuring both arm blood pressure is a valuable clinical parameter during the in-hospital stay and careful attention should be paid to perioperative blood pressure, which may reduce the risk of procedural complications. A difference in IASBPD of ≥ 20 mmHg had a significant correlation with both subclavian artery stenosis, and subclavian artery stenosis was associated with perioperative stroke, preoperative symptoms and vertebral artery stenosis. However, we could not conclude any correlation with carotid artery stenosis, and subclavian artery stenosis and IASBPD. Those groups of patients with more than 20 mmHg of IASBPD should definitely be investigated for subclavian artery stenosis.

**Table:** 

Authors' roles & responsibilities	
SBD	Design, analysis, writing; final approval of the version to be published
SMY	Design, writing; final approval of the version to be published
MKD	Design; final approval of the version to be published
FK	Analysis; final approval of the version to be published
HTK	Analysis; final approval of the version to be published
